# Protein‐Nanocaged Selenium Induces t(8;21) Leukemia Cell Differentiation via Epigenetic Regulation

**DOI:** 10.1002/advs.202300698

**Published:** 2023-10-27

**Authors:** Long Fang, Ruofei Zhang, Lin Shi, Jiaying Xie, Long Ma, Yili Yang, Xiyun Yan, Kelong Fan

**Affiliations:** ^1^ Savaid Medical School University of Chinese Academy of Sciences Beijing 100049 China; ^2^ CAS Engineering Laboratory for Nanozyme Key Laboratory of Biomacromolecules Institute of Biophysics Chinese Academy of Sciences Beijing 100101 China; ^3^ Department of Hematology Peking University International Hospital Beijing 102206 China; ^4^ China Regional Research Centre International Centre of Genetic Engineering and Biotechnology Taizhou 212200 China; ^5^ Nanozyme Medical Center School of Basic Medical Sciences Zhengzhou University Zhengzhou 450052 China

**Keywords:** AML1‐ETO, oncoprotein degradation, protein nanocage, selenium nanoparticle, t(8;21) leukemia

## Abstract

The success of arsenic in degrading PML‐RARα oncoprotein illustrates the great anti‐leukemia value of inorganics. Inspired by this, the therapeutic effect of inorganic selenium on t(8; 21) leukemia is studied, which has shown promising anti‐cancer effects on solid tumors. A leukemia‐targeting selenium nanomedicine is rationally built with bioengineered protein nanocage and is demonstrated to be an effective epigenetic drug for inducing the differentiation of t(8;21) leukemia. The selenium drug significantly induces the differentiation of t(8;21) leukemia cells into more mature myeloid cells. Mechanistic analysis shows that the selenium is metabolized into bioactive forms in cells, which drives the degradation of the AML1‐ETO oncoprotein by inhibiting histone deacetylases activity, resulting in the regulation of AML1‐ETO target genes. The regulation results in a significant increase in the expression levels of myeloid differentiation transcription factors PU.1 and C/EBPα, and a significant decrease in the expression level of C‐KIT protein, a member of the type III receptor tyrosine kinase family. This study demonstrates that this protein‐nanocaged selenium is a potential therapeutic drug against t(8;21) leukemia through epigenetic regulation.

## Graphical abstract

A protein‐caged selenium nanodrug is developed, which selectively targets t(8;21) leukemia cells by binding to TfR1 and VLA‐4 receptors. Upon entering the cells, the nanodrug metabolizes into selenocysteine, which inhibits HDACs activity, leading to histone acetylation and AML1‐ETO degradation. Thus, the protein‐caged selenium nanodrug promotes the differentiation of t(8;21) leukemia cells.

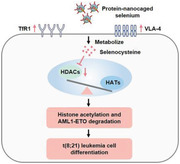

## 1 Introduction

Genome abnormalities caused by chromosomal translocations play a key role in the development of leukemia.^[^ ^1^ ^]^ The t(8;21) chromosomal translocation generated a chimeric gene encoding AML1‐ETO oncoprotein that contains the N‐terminal DNA‐binding domain of acute myeloid leukemia 1 (AML1) gene and nearly full‐length eight‐twenty one (ETO) gene. The translocation is reported in 12–20% of AML patients and 40–80% of AML patients with French‐American‐British type M2 (FAB‐M2) morphology.^[^ ^2^ ^]^ The AML1‐ETO fusion protein induces leukemogenesis by disrupting the differentiation of normal hematopoietic cells and alternating cell proliferation.^[^ ^2^, ^3^ ^]^ Studies have shown that AML1‐ETO inhibits the expression of C/EBPα^[^ ^4^ ^]^ and PU.1,^[^ ^5^ ^]^ and up‐regulates the receptor for stem cell growth factor protein C‐KIT.^[^ ^6^ ^]^ It has also been found that AML1‐ETO interacts with nuclear receptor corepressor (NCoR) and mammalian Sin3 (mSin3), which in turn binds to histone deacetylases (HDACs), leading to repression of AML1 target genes transcription.^[^ ^7^ ^]^ Therefore, AML1‐ETO is a promising target for anti‐t(8;21) leukemia therapy. It is worth noting that arsenic trioxide is an effective therapeutic for promyelocytic leukemia with t(15;17) translocation that produces the fusion protein promyelocytic leukemia (PML)‐retinoic acid receptor‐α (RARα). It has been shown that arsenic trioxide directly binds to cysteine residues in the zinc fingers located in the RBCC domain of PML, triggering the SUMOylation and degradation of the fusion protein PML‐RARα.^[^ ^8^ ^]^ Therefore, looking for novel inorganics that effectively treat hematological tumors via targeted degradation of oncoproteins (e.g., AML1‐ETO) may be a promising strategy to defeat leukemia.

Selenium, an essential trace element in humans,^[^ ^9^ ^]^ exhibited a good therapeutic effect on a variety of solid tumor cells (HT29, MCF‐7, A549, HepG‐2) when delivered in nanoparticles.^[^ ^10^ ^]^ The selenium nanoparticles are also capable of inducing apoptosis of leukemia cells.^[^ ^11^ ^]^ However, the underlying mechanisms of the cytotoxic actions of the selenium nanoparticles remain largely elusive, although ROS has been attributed as a mediator under certain circumstances.^[^ ^12^ ^]^ It has also been reported that clinical translations of the selenium nanoparticles were severely hindered by their heterogeneous sizes and limited targeting capacity.^[^ ^12^, ^13^ ^]^

Ferritin, a natural carrier of inorganic minerals, is composed of heavy chain and light chain subunits in different proportions to form a 24‐subunit spherical protein nanocage.^[^ ^14^ ^]^ Using ferritin as a template, uniform and dispersed nanoparticles can be synthesized through biomineralization. Of note, ferritin specifically binds to transferrin receptor 1 (TfR1), which is often expressed highly in cells of solid tumors and leukemias.^[^ ^15^ ^]^ Ferritin nanocage may also recognize various targets by genetic or chemical modification to display functional ligands on its surface. It has been shown recently that ferritin nanocages loaded with different metals have good targeting and pharmacokinetic properties in animal models.^[^ ^16^ ^]^

**Scheme 1:**
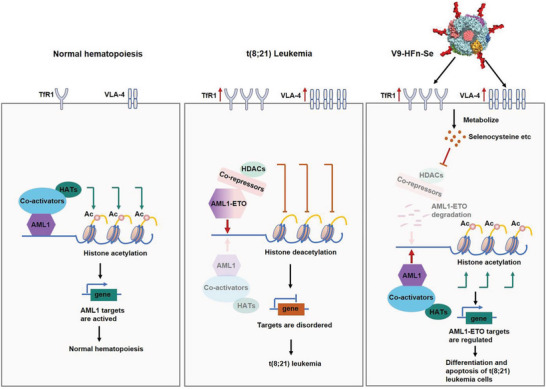
The mechanisms of V9‐HFn‐Se targeting and inducing the differentiation of t(8;21) leukemia cells. In normal hematopoietic cells, AML1 binds coactivators and recruits histone acetyltransferases (HATs) to induce histone acetylation (Ac), which in turn initiates the transcription of AML1 target genes and regulates normal hematopoiesis. In t(8;21) leukemia, the AML1‐ETO oncoprotein takes up the transactivation sites of AML1 and recruits co‐repressors and HDACs, resulting in histone deacetylation and subsequently changes in gene expression and hematopoietic differentiation programs. TfR1 and VLA‐4 are highly expressed in acute myeloid leukemia cells. The dual‐targeted V9‐HFn‐Se that was designed based on these two targets efficiently induces differentiation of t(8;21) leukemia cells. V9‐HFn‐Se enters leukemia cells and is metabolized to generate selenocysteine, which inhibits the activity of HDACs, leading to the degradation of the AML1‐ETO oncoprotein and the regulation of target genes.

In the present study, we designed and prepared a ferritin‐based nanomedicine (V9‐HFn‐Se) that expressed ligand for very late antigen 4 (VLA‐4) on the surface and contained selenium inside the cavity. We found that V9‐HFn‐Se selectively killed leukemia cells expressing VLA‐4,^[^ ^17^ ^]^ and TfR1. The leukemia‐targeting V9‐HFn‐Se induced effectively the degradation of AML1‐ETO and altered the expression of its target genes in t(8;21) leukemia cells. These changes result in the differentiation and apoptosis of the leukemia cells (**Scheme** **1**), suggesting that V9‐HFn‐Se is a promising therapeutics for the differentiation therapy of t(8;21) leukemia.

## 2 Results

### 2.1 HFn‐Se Inhibits Preferentially t(8;21) Leukemia Cells

**Figure 1:**
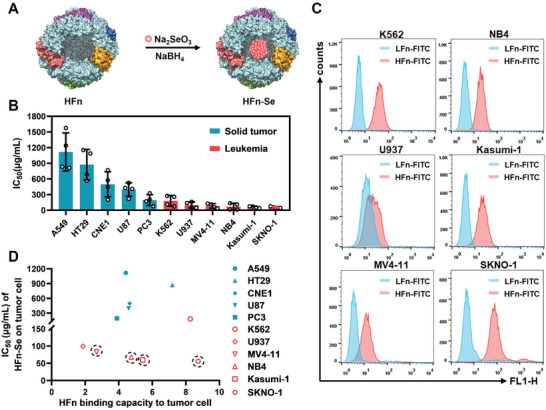
Synthesis of HFn‐Se and its effects on cancer cells. A) Schematic diagram of the preparation of HFn‐Se. B) The half maximal inhibitory concentration (IC_50_) of HFn‐Se on different tumor cells (*n* = 4). C) Binding capacity of HFn on different leukemia cells. Light chain ferritin (LFn) was chosen as a negative control, which has similar structural features to HFn but does not significantly bind to tumor cells. D) The correlation between the half maximal inhibitory concentration (IC_50_) of HFn‐Se and the binding capacity of HFn to different tumor cells. The binding capacity of HFn was evaluated by the fluorescence intensity ratio of HFn‐FITC to LFn‐FITC bound to tumor cells. The data represent mean ± SD.

Mono‐dispersed spherical selenium nanoparticles (HFn‐Se) were synthesized via in situ reduction of Na_2_SeO_3_ in the cavity of recombinant human ferritin heavy chain (HFn) (**Figure** **1**A; Figure S1A,B, Supporting Information). The effects of HFn‐Se on the viability of several lines of tumor cells were examined. The leukemia cell lines SKNO‐1, Kasumi‐1, NB4, and MV4‐11 were markedly more sensitive to HFn‐Se than solid tumor cells. As shown in Figure 1B, the IC_50_ of HFn‐Se for SKNO‐1 and Kasumi‐1 were 55.98 and 59.51 µg mL^−1^, respectively, whereas that of A549 was 1117 µg mL^−1^. Since HFn targets tumor cells via binding to TfR1, we analyzed the relationship between HFn‐Se sensitivity and binding capacity of HFn in different lines of cells. The results show that the sensitivity of both solid and leukemia cells to HFn‐Se was independent of HFn binding, suggesting that the intrinsic mechanisms in leukemia cells make them more sensitive to HFn‐Se (Figure 1B–D; Figure S1C, Supporting Information).

### 2.2 V9‐HFn‐Se Targets t(8;21) Leukemia Cells through TfR1 and VLA‐4

**Figure 2:**
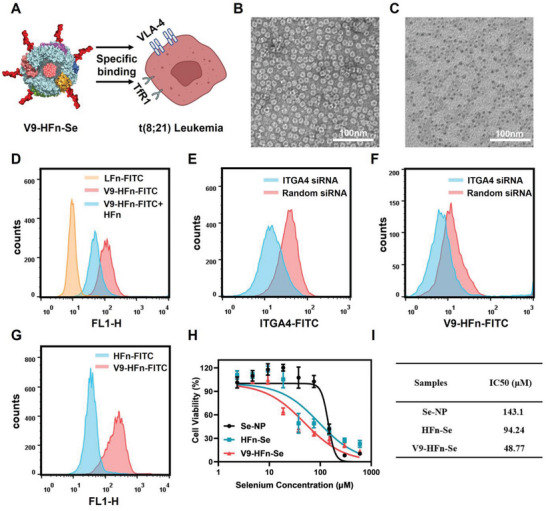
Preparation of V9‐HFn‐Se and its specific binding to t(8;21) leukemia cells. A) Schematic illustration of V9‐HFn‐Se dual‐targeting binding to TfR1 and VLA‐4 receptors on t(8;21) leukemia cells. B,C) TEM images of the ferritin shell and the selenium core of V9‐HFn‐Se. Scale bar = 100 nm. D) Flow cytometric analysis of the binding specificity of V9‐HFn to Kasumi‐1 cells. E) Flow cytometric analysis of ITGA4 expression in Kasumi‐1 cells after knockdown with siRNA. F) Flow cytometric analysis of the binding specificity of V9‐HFn to ITGA4‐knockdown Kasumi‐1 cells. G) Flow cytometric analysis of the comparison between the binding specificities of V9‐HFn and HFn to Kasumi‐1 cells. H) Cell viability of Kasumi‐1 cells treated with selenium nanoparticles (Se‐NP), HFn‐Se, and V9‐HFn‐Se, respectively (*n* = 3), and I) the corresponding half maximum inhibitory concentration (IC_50_).

Relapse after chemotherapy is a significant challenge in the treatment of acute myeloid leukemia.^[^ ^18^ ^]^ It has been shown that VLA‐4 was highly expressed in acute myeloid leukemia cells and was closely related to chemoresistance.^[^ ^17a^ ^]^ Therefore, a VLA‐4‐targeting peptide (V9) was genetically displayed on the surface of HFn‐Se to generate V9‐HFn‐Se that could target tumor cells through binding to TfR1 and VLA‐4 (**Figure** **2**A). Compared to HFn, V9‐HFn has an increased molecular weight (Figure S2A, Supporting Information). The sizes of HFn and V9‐HFn were also measured by dynamic light scattering (DLS). The average diameter of HFn and V9‐HFn were 14 ± 3.431 and 16.97 ± 3.636 nm, respectively (Figure S2B,C, Supporting Information). These results indicated a successful display of the VLA‐4‐targeted peptides on the protein surface. Similar to HFn‐Se, V9‐HFn‐Se retains a mono‐dispersed core‐shell spherical structure with 530 selenium atoms loaded in one V9‐HFn (Figure 2B,C; Figure S3A,B, Supporting Information). As shown in Figure 2D, FITC‐labeled V9‐HFn bound to Kasumi‐1 cells efficiently, which was blocked markedly by unlabeled HFn. However, V9‐HFn‐FITC has no targeting effect on human normal HUVEC cells (Figure S4A, Supporting Information). We also knocked down ITGA4 (CD49D, a subunit of VLA‐4) in Kasumi‐1 cells using a siRNA (Figure 2E). As shown in Figure 2F, the binding of FITC‐labeled V9‐HFn to Kasumi‐1 cells with ITGA4 knockdown was also notably attenuated. These results indicated that V9‐HFn targeted the cells through both TfR1 and VLA‐4. Further analyses found that V9‐HFn exhibited stronger binding for Kasumi‐1 than that of HFn (Figure 2G), and V9‐HFn‐Se reduced cell viability more efficiently than that of Se‐NP and HFn‐Se (Figure 2H,I). V9‐HFn did not significantly affect the viability of HUVECs and Kasumi‐1 cells (Figures S4B and S5, Supporting Information). To further validate the targeting of V9‐HFn‐Se, we detected the expression levels of TfR1 and ITGA4 in different leukemia cells. Western blot analysis revealed low expression of TfR1 and ITGA4 in U937 cells, while high expression of TfR1 and ITGA4 in Kasumi‐1 cells (Figure S6A–D, Supporting Information). Compared with U937 cells, V9‐HFn‐Se has a more significant sensitivity to Kasumi‐1 (Figure S6E, Supporting Information). Moreover, the targeted efficiency of V9‐HFn to Kasumi‐1 cells was not affected by the Se loading (Figure S7, Supporting Information). Taken together, these data indicated that V9‐HFn‐Se acts on Kasumi‐1 more effectively than Se‐NP and HFn‐Se through interacting with both TfR1 and VLA‐4.

### 2.3 V9‐HFn‐Se Induces Differentiation and Apoptosis of t(8;21) Leukemia Cells

**Figure 3:**
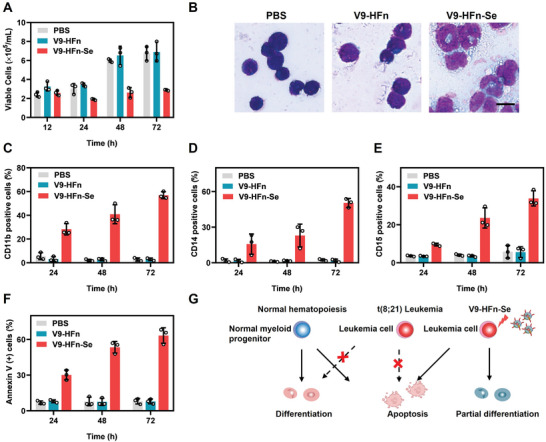
V9‐HFn‐Se inhibits the growth of t(8;21) leukemia cells by inducing differentiation and apoptosis. A) Effects of V9‐HFn or V9‐HFn‐Se on the growth of Kasumi‐1 cells (*n* = 3). B) Morphological changes of Kasumi‐1 cells after exposure to 60 µg mL^−1^ V9‐HFn or V9‐HFn‐Se for 6 days. Kasumi‐1 treated with PBS, V9‐HFn, and V9‐HFn‐Se and stained with Wright's stain were analyzed under a microscope (20×). Scale bar = 20 µm. C–E) Flow cytometric analysis of CD11b, CD14, and CD15 expression in Kasumi‐1 cells treated with 270 µg mL^−1^ V9‐HFn or V9‐HFn‐Se in a time‐dependent manner (*n* = 3). F) Annexin‐V staining of Kasumi‐1 cells treated with 270 µg mL^−1^ V9‐HFn or V9‐HFn‐Se for the indicated time (*n* = 3). G) Schematic diagram of differentiation and apoptosis of Kasumi‐1 cells induced by V9‐HFn‐Se. The data represent mean ± SD.

The ability of V9‐HFn‐Se to efficiently reduce the growth of Kasumi‐1 cells (**Figure** **3**A) propelled us to explore the underlying mechanisms. After being exposed to V9‐HFn‐Se, Kasumi‐1 cells were enlarged and showed more mature morphology (Figure 3B). V9‐HFn‐Se‐treated Kasumi‐1 also have increased levels of CD11b, CD14, and CD15 in a time‐dependent manner (Figure 3C–E), indicating that V9‐HFn‐Se induces the differentiation of Kasumi‐1 cells into myeloid cells. Interestingly, V9‐HFn also exhibited good targeting to SKNO‐1, another t(8;21) leukemia model cell line (Figure S8, Supporting Information). V9‐HFn‐Se also increased the expression levels of CD11b, CD14, and CD15 in SKNO‐1 cells in a time‐dependent manner (Figure S9A–C, Supporting Information).

We further examined the ability of V9‐HFn‐Se to induce apoptosis in t(8;21) leukemia cells. Annexin V‐positive t(8;21) leukemia cells increased upon V9‐HFn‐Se treatment (Figure 3F; Figure S9D, Supporting Information). JC‐1 staining revealed a significant decrease in mitochondrial membrane potential (Figure S10, Supporting Information), indicating that V9‐HFn‐Se induces apoptosis of Kasumi‐1 cells effectively. Of note, V9‐HFn‐Se‐treated cells were mostly arrested in the S and G2/M phases of the cell cycle (Figure S11, Supporting Information). Thus, V9‐HFn‐Se inhibits the growth of t(8;21) leukemia cells by triggering differentiation as well as apoptosis (Figure 3G). Interestingly, arsenic trioxide, which induces apoptosis and partial differentiation of acute promyelocytic leukemia, mainly caused apoptosis in Kasumi‐1 cells (Figure S12A,B, Supporting Information).

### 2.4 V9‐HFn‐Se Causes AML1‐ETO Fusion Protein Degradation

**Figure 4:**
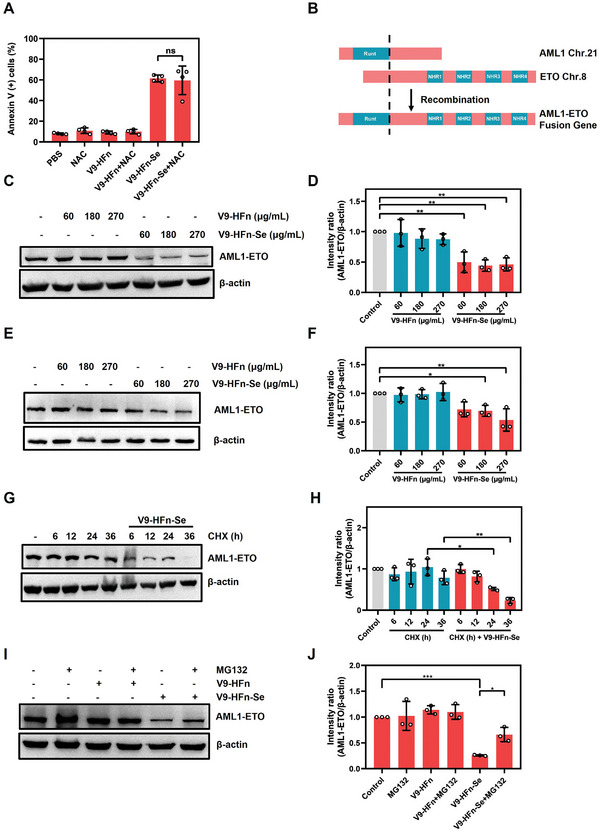
V9‐HFn‐Se induces AML1‐ETO degradation. A) The percentage of Annexin V‐positive cells in Kasumi‐1 cells after V9‐HFn‐Se treatment in the presence or absence of NAC (*n* = 4). B) Schematic diagram of AML1‐ETO fusion gene. C,D) Immunoblotting and quantification of AML1‐ETO protein level under indicated concentrations of V9‐HFn or V9‐HFn‐Se treatment for 48 h in Kasumi‐1 (*n* = 3). The protein intensity of AML1‐ETO was quantified and represented as relative protein levels normalized to β‐actin. E,F) Immunoblotting and quantification of AML1‐ETO protein level in SKNO‐1 cells treated with different concentrations of V9‐HFn or V9‐HFn‐Se for 48 h (*n* = 3). Protein intensity of AML1‐ETO was quantified and represented as relative protein levels normalized to β‐actin. G,H) Immunoblotting and quantification of AML1‐ETO level in Kasumi‐1 cells treated with 10 µg mL^−1^ CHX with or without V9‐HFn‐Se for the indicated periods (*n* = 3). Protein intensity of AML1‐ETO was quantified and represented as relative protein levels normalized to β‐actin. I,J) Immunoblotting and quantification of AML1‐ETO level in V9‐HFn‐Se‐treated Kasumi‐1 cells for 24 h with the addition of 2 µm MG132 for the last 6 h (*n* = 3). Protein intensity of AML1‐ETO was quantified and represented as relative protein levels normalized to β‐actin. ^*^ *p* < 0.05, ^**^ *p* < 0.01, ^***^ *p* < 0.001. One‐way ANOVA with Dunnett's (D,F) or Tukey's test (A,H,J) was performed. The data represent mean ± SD.

It has been shown that selenium nanoparticles induced apoptosis of solid tumor cells by generating ROS.^[^ ^12^ ^]^ Although V9‐HFn‐Se caused a significant increase of ROS in Kasumi‐1 cells (Figure S13, Supporting Information), we found that ROS scavenger N‐acetylcysteine (NAC) did not prevent V9‐HFn‐Se‐induced apoptosis (**Figure** **4**A). Interestingly, AML1‐ETO fusion protein, which contains the N‐terminal DNA‐binding domain of AML1 and nearly full‐length ETO resulted from t(8;21) translocation (Figure 4B), was reduced by V9‐HFn‐Se dose‐dependently at protein level (Figure 4C–F). As V9‐HFn‐Se did not alter the level of AML1‐ETO mRNA (Figure S14, Supporting Information), it likely acted by inducing the fusion protein degradation. We explored the effect of V9‐HFn‐Se on the stability of AML1‐ETO fusion protein in the presence of a protein synthesis inhibitor, cycloheximide (CHX), and the results showed that V9‐HFn‐Se significantly induced the degradation of AML1‐ETO (Figure 4G,H). It has been suggested that the degradation of AML1‐ETO may result from the cell death process, particularly the activation of caspase‐3.^[^ ^19^ ^]^ However, V9‐HFn‐Se‐induced AML1‐ETO degradation was not prevented by the potent caspase‐3 inhibitor Z‐DQMD‐FMK (Figure S15A,B, Supporting Information), indicating that the degradation does not result from the cell death pathway. To analyze whether proteasomes participate in the V9‐HFn‐Se‐induced AML1‐ETO degradation, we evaluated the effect of these enzymes using proteasome chemical inhibitors. As shown in Figure 4I,J, proteasome inhibitor MG132 partially rescued V9‐HFn‐Se mediated reduction of AML1‐ETO protein level in Kasumi‐1 cells, suggesting that V9‐HFn‐Se degrades AML1‐ETO oncoprotein depends on proteasome pathway. It is conceivable that AML1‐ETO degradation altered the cellular transcription program that led to the death of leukemia cells.

### 2.5 V9‐HFn‐Se Alters the Transcription Program of AML1‐ETO

**Figure 5:**
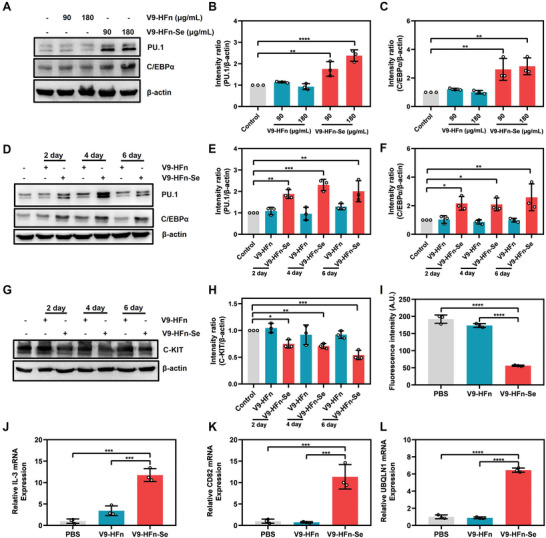
V9‐HFn‐Se affects the levels of C/EBPα, PU.1, and C‐KIT, and induces the expression of genes inhibited by AML1‐ETO. Immunoblotting and quantification of PU.1 and C/EBPα protein level in Kasumi‐1 cells treated with indicated concentrations of V9‐HFn or V9‐HFn‐Se (A–C) and for different times (D–F) (*n* = 3). Protein intensity of PU.1 and C/EBPα were quantified and represented as relative protein levels normalized to β‐actin. G,H) Immunoblotting and quantification of C‐KIT protein level in Kasumi‐1 cells at indicated times after V9‐HFn or V9‐HFn‐Se treatment (*n* = 3). Protein intensity of C‐KIT was quantified and represented as relative protein levels normalized to β‐actin. I) Flow cytometric analysis of C‐KIT expression in Kasumi‐1 cells treated with V9‐HFn or V9‐HFn‐Se for 48 h (*n* = 3). The IL‐3 (J), CD82 (K), and UBQLN1 (L) expression in Kasumi‐1 cells treated with 90 µg mL^−1^ V9‐HFn or V9‐HFn‐Se for 48 h as measured by qPCR. ^*^ *p* < 0.05, ^**^ *p* < 0.01, ^***^ *p* < 0.001, ^****^ *p* < 0.0001. One‐way ANOVA with Dunnett's test (B,C,E,F,H) or Tukey's test (I,J,K,L) was performed. The data represent mean ± SD.

It has been reported that AML1‐ETO inhibits the expression of PU.1 and C/EBPα, two important transcription factors for the differentiation of myeloid cells, thereby preventing the differentiation of t(8,21) type leukemia cells.^[^ ^4^, ^5^ ^]^ Interestingly, V9‐HFn‐Se increased the expressions of PU.1 and C/EBPα in Kasumi‐1 cells (**Figure** **5**A–F). It has also been shown that t(8;21) leukemia had C‐KIT overexpression and/or gain‐of‐function mutation,^[^ ^6^ ^]^ which presumably contributes to the development of the disease and is a promising therapeutic target. In Kasumi‐1 cells treated with V9‐HFn‐Se, the expression of C‐KIT was markedly reduced in a time‐dependent manner (Figure 5G–I). Of note, AML1‐ETO recruits the nuclear receptor co‐repressor to form an NCoR‐mSin3‐HDAC complex that inhibits the expression of target genes such as IL‐3, CD82, and UBQLN1.^[^ ^2^, ^20^ ^]^ As shown in Figure 5J–L, V9‐HFn‐Se increased significantly the expression of IL‐3, CD82, and UBQLN1 as measured by qPCR. Taken together, these results demonstrated that V9‐HFn‐Se regulates the transcription of many target genes of AML1‐ETO.

### 2.6 V9‐HFn‐Se Inhibits HDACs Activity, Leading to Histone Acetylation and Chromatin Remodeling

**Figure 6:**
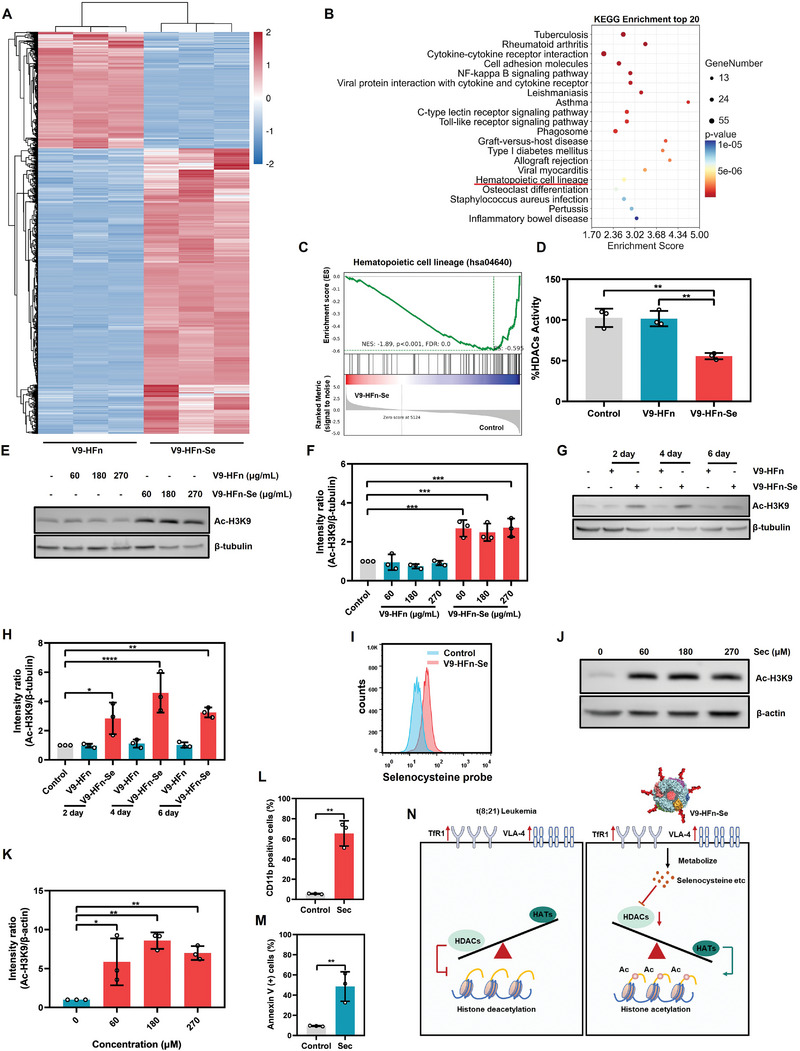
V9‐HFn‐Se induces histone acetylation and chromatin remodeling. A) RNA‐seq analysis was performed in Kasumi‐1 cells treated with 90 µg mL^−1^ V9‐HFn or V9‐HFn‐Se for 72 h. The heatmap shows genes whose expression is significantly changed by more than 1.5‐fold. B) The top 20 functionally enriched KEGG pathways of differentially expressed genes in V9‐HFn‐Se versus Control. C) The KEGG‐enrichment plots of representative gene sets from hematopoietic cell lineage pathway based on gene set enrichment analysis (GSEA). D) Measurement of HDACs activity in Kasumi‐1 cells treated with 90 µg mL^−1^ V9‐HFn or V9‐HFn‐Se for 72 h (n = 3). Immunoblotting and quantification of H3K9 acetylation level in Kasumi‐1 cells treated with indicated concentrations of V9‐HFn or V9‐HFn‐Se (E,F) and for indicated times (G,H) (*n* = 3). Protein intensity of Ac‐H3K9 was quantified and represented as relative protein levels normalized to β‐tubulin. I) Flow cytometric analysis of selenocysteine in Kasumi‐1 cells treated with 30 µg mL^−1^ V9‐HFn‐Se for 48 h. J,K) Immunoblotting and quantification of acetylated histone H3K9 levels in Kasumi‐1 cells treated with different concentrations of selenocysteine (*n* = 3). Protein intensity of Ac‐H3K9 was quantified and represented as relative protein levels normalized to β‐actin. L) Flow cytometric analysis of CD11b expression in Kasumi‐1 cells treated with selenocysteine for 72 h (*n* = 3). M) The percentage of Annexin V‐positive Kasumi‐1 cells after being treated with selenocysteine for 72 h (*n* = 3). N) Schematic illustration of the inhibition of HDACs activity by V9‐HFn‐Se. ^*^ *p* < 0.05, ^**^ *p* < 0.01, ^***^ *p* < 0.001, ^****^ *p* < 0.0001. One‐way ANOVA with Tukey's test (D) or Dunnett's test (F,H,K) was performed. Two‐tailed unpaired Student's *t*‐test (L,M) was performed. The data represent mean ± SD.

To further explore the molecular mechanism of V9‐HFn‐Se against t(8;21) leukemia cells, we conducted an RNA‐seq analysis of Kasumi‐1 cells treated with V9‐HFn or V9‐HFn‐Se. V9‐HFn‐Se treatment altered the levels of 2280 gene transcripts (**Figure** **6**A). KEGG enrichment analysis showed that V9‐HFn‐Se‐induced alterations were enriched in hematopoietic cell lineage‐related genes (Figure 6B,C). Several studies have shown that histone acetylation induced by HDAC inhibitors promotes the differentiation of t(8;21) leukemia.^[^ ^21^ ^]^ We found that HDAC inhibitors trichostatin A and panobinostat induced differentiation and apoptosis of t(8; 21) leukemia cells as well as reducing the level of AML1‐ETO protein (Figures S16A–D and S17A–D, Supporting Information). Further, V9‐HFn‐Se effectively inhibited the activity of HDACs (Figure 6D) and increased H3K9 acetylation (Ac‐H3K9) in Kasumi‐1 cells (Figure 6E–H). Interestingly, like selenium nanoparticles, V9‐HFn‐Se was metabolized intracellularly to form selenocysteine (Sec) that could be measured with the near‐infrared fluorescent probes^[^ ^22^ ^]^ (Figure 6I). H3K9 acetylation levels were also increased markedly in Kasumi‐1 cells exposed to selenocysteine (Figure 6J,K). After 72 h treatment with selenocysteine, Kasumi‐1 cells underwent differentiation and apoptosis (Figure 6L,M). Therefore, it is likely that V9‐HFn‐Se metabolite selenocysteine inhibits HDACs and causes AML1‐ETO degradation, which leads to H3K9 acetylation, chromatin remodeling, change of gene expression profile, and differentiation and apoptosis of t(8;21) leukemia cells^[^ ^21a,d^ ^]^ (Figure 6N).

### 2.7 Specificity and Efficacy of V9‐HFn‐Se in the Simulated In Vivo Microenvironment

**Figure 7:**
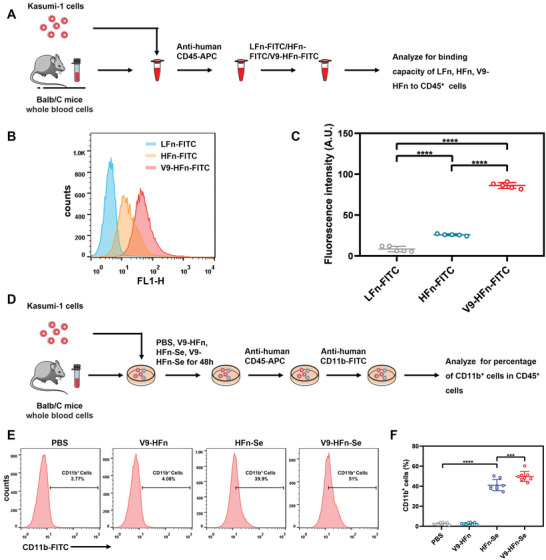
V9‐HFn‐Se targets and induces t(8;21) leukemia cell differentiation in an in vivo‐like microenvironment model. A) Schematic diagram of LFn, HFn, V9‐HFn targeting simulation test in vivo. B) Flow cytometric analysis and C) quantification of the binding capacity of LFn, HFn, V9‐HFn to human leukemia cells in simulated the microenvironment in vivo (*n* = 5). D) The schematic diagram of V9‐HFn, HFn‐Se, V9‐HFn‐Se induced differentiation of human leukemia cells in simulated in vivo microenvironment. E) Flow cytometric analysis and F) quantification of V9‐HFn, HFn‐Se, V9‐HFn‐Se induced differentiation of human leukemia cells in a simulated in vivo microenvironment (*n* = 8). ^***^ *p* < 0.001, ^****^ *p* < 0.0001. One‐way ANOVA with Tukey's test (C,F) was performed. The data represent mean ± SD.

Since the mouse model of human t(8; 21) leukemia cannot be effectively established at present,^[^ ^23^ ^]^ we evaluated the specificity of V9‐HFn by mixing the whole blood of Balb/C mice with Kasumi‐1 cells in vitro to simulate the blood microenvironment (**Figure** **7**A). An anti‐human CD45‐APC antibody was used to distinguish Kasumi‐1 cells from mouse blood cells. Compared with LFn‐FITC, HFn‐FITC and V9‐HFn‐FITC both bound to CD45^+^ cells efficiently, and V9‐HFn‐FITC showed the highest binding (Figure 7B,C). We then evaluated the effect of V9‐HFn‐Se on Kasumi‐1 cell differentiation in the same system (Figure 7D). Following incubation with V9‐HFn‐Se for 48 h, while HFn‐Se and V9‐HFn‐Se both effectively induced CD11b expression on CD45^+^ cells, V9‐HFn‐Se was a more potent inducer (Figure 7E,F). These data indicate that V9‐HFn‐Se possesses good specificity and efficacy in the simulated in vivo microenvironment, and TfR1 and VLA‐4 both contribute to the specific targeting.

### 2.8 V9‐HFn‐Se Induces Differentiation in Human Primary t(8;21) AML Cells

**Figure 8:**
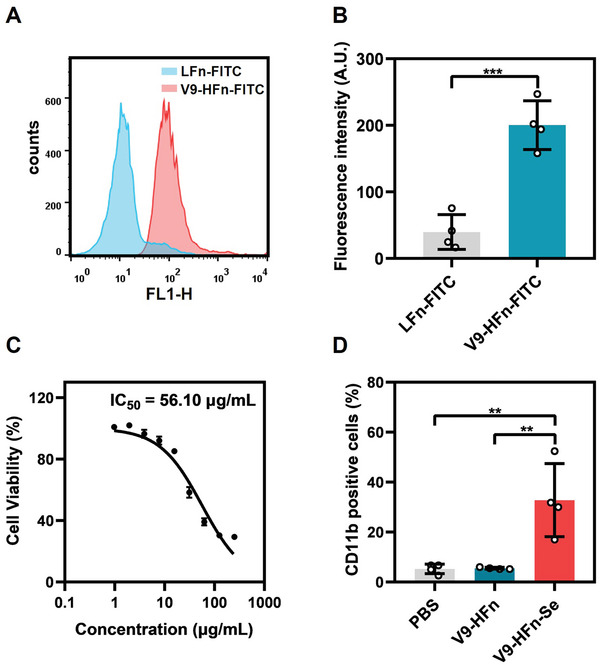
V9‐HFn‐Se targets and induces differentiation in human primary t(8;21) leukemia cells. A) Flow cytometric analysis and B) quantification of the binding specificity of V9‐HFn to human primary t(8;21) leukemia cells (*n* = 4). C) Cell viability of human primary t(8;21) leukemia cells treated with V9‐HFn‐Se. D) Flow cytometric analysis of CD11b expression in human primary t(8;21) leukemia cells treated with 60 µg mL^−1^ V9‐HFn or V9‐HFn‐Se for 48 h (*n* = 4). ^**^ *p* < 0.01, ^***^ *p* < 0.001. Two‐tailed unpaired Student's *t*‐test (B) was performed. One‐way ANOVA with Tukey's test (D) was performed. The data represent mean ± SD.

We then conducted experiments to analyze whether V9‐HFn‐Se possesses any therapeutic effect on the human primary t(8;21) leukemia cells. The bone marrow samples were collected from four t(8;21) AML patients and the leukemia cells were then obtained by ficoll isolation. Using LFn‐FITC as a control, the results showed a substantial binding of V9‐HFn‐FITC to the primary t(8;21) leukemia cells (**Figure** **8**A,B). Moreover, the primary t(8;21) leukemia cells demonstrated high sensitivity and a propensity for differentiation upon the induction of V9‐HFn‐Se (Figure 8C,D). We also evaluated the cytotoxicity of V9‐HFn‐Se on human peripheral blood mononuclear cells. The results showed that V9‐HFn‐Se exhibited no obvious cytotoxicity to human peripheral blood mononuclear cells (Figure S18, Supporting Information), indicating that it has a good safety profile for human normal blood cells.

## 3 Discussion

The remarkable success of arsenic trioxide in the treatment of acute promyelocytic leukemia raised the question of whether other inorganics could also have a potent and therapeutic anti‐leukemia effect. Here, we found that selenium loaded in ferritin nanocage triggered a differentiation and apoptotic effect on t(8;21) leukemia cells attributed to the degradation of the AML1‐ETO oncoprotein and the regulation of its target genes. This study demonstrates that V9‐HFn‐Se may be a promising inorganic agent for the treatment of t(8;21) leukemia through epigenetic modulation.

Tumor‐targeting is a key factor that severely affects the pharmacokinetics and therapeutic efficacy of nanomedicines, especially in the treatment of hematological tumors. Selenium nanoparticles have been shown to exhibit impressive anti‐tumor effects. However, the clinical translations are severely limited by their size heterogeneity, limited targeting ability, and toxic side effects. Thus, we loaded selenium to the ferritin nanocage with uniform size and selective binding to TfR1 and VLA‐4 that are expressed on many tumor cells. Compared with Se‐nanoparticles and HFn‐Se, the genetically engineered V9‐HFn‐Se exhibited a stronger anti‐t(8;21) leukemia cell effect with a lower IC_50_. V9‐HFn‐Se also showed good targeting specificity and therapeutic efficacy in a simulated in vivo microenvironment. We speculated that V9‐HFn‐Se recognizes and enters t(8;21) leukemia cells more efficiently compared to Se‐nanoparticles and HFn‐Se, thus exerting a stronger intracellular effect. These results suggest that further studies in t(8;21) animals or patients are warranted to validate the anti‐leukemic potential of V9‐HFn‐Se. In addition, the therapeutic effect of V9‐HFn‐Se on other leukemia types is also expected because both TfR1 and VLA‐4 are widely highly expressed markers in acute myeloid leukemia.

Previous studies suggest that the anti‐cancer effect of selenium nanoparticles on solid tumors was mainly mediated by ROS generation. It is worth noting that, although V9‐HFn‐Se significantly induced the up‐regulation of ROS level in t(8;21) leukemia cells, the increased ROS did not contribute significantly to the anti‐leukemia effect. We found that V9‐HFn‐Se induced the differentiation of t(8;21) leukemia cells and subsequent apoptotic cell death by causing the degradation of the AML1‐ETO oncoprotein without affecting its transcription. Moreover, V9‐HFn‐Se‐induced AML1‐ETO degradation does not depend on the caspase‐3‐mediated cell death process, but depends on the proteasome degradation pathway. Interestingly, it has been shown that HDAC inhibitors affect the binding of HSP90 to AML1‐ETO, thereby triggering the proteasome pathway to degrade AML1‐ETO. It is likely that V9‐HFn‐Se‐mediated degradation of AML1‐ETO also depends on the ubiquitination proteasome system.^[^ ^21^, ^24^ ^]^

It has been reported that the degradation of AML1‐ETO altered the expression of multiple target genes such as BCL‐2, MPO, GM‐CSF, and IL‐3,^[^ ^19c^ ^]^ which appear to participate in cell death program. Interestingly, we found in the present study that V9‐HFn‐Se also increases the expression of key differentiation‐related transcription factor PU.1, C/EBPα, and decreases the expression of stem cell growth factor receptor C‐KIT, along with cell differentiation. Of note, while AML1 recognizes specific sequences and recruits HAT and co‐activator, AML1‐ETO brings co‐repressor and HDAC to the same site. It is conceivable that the replacement of HAT with HDAC causes histone deacetylation, leading to alterations of chromatin structure and gene expression,^[^ ^25^ ^]^ which are responsible for cell transformation. We found in the investigation that intracellular selenium, likely selenocysteine,^[^ ^26^ ^]^ may act through inhibiting HDACs, which not only increases H3K9 acetylation to alter gene expression, but also degradation of AML1‐ETO fusion proteins and further reduction of histone deacetylation at a specific site, which result in the restore of gene expression, cell differentiation, and apoptosis. It is interesting and important to further explore how inhibition of HDACs leads to AML1‐ETO and how the fusion protein gets degraded.

Taken together, these data indicated that V9‐HFn‐Se can deliver selenium effectively and selectively to leukemia cells, and open up a novel avenue for the treatment of t(8;21) leukemia.

## 4 Experimental Section

### Reagents

Z‐DQMD‐FMK, MG132, Trichostatin A, and Cycloheximide were obtained from MedChem Express (Monmouth Junction, NJ, USA). Sodium arsenite and Sodium selenite were obtained from Sigma (St Louis, MO, USA). N‐Acetyl‐L‐Cysteine was obtained from Aladdin (Shanghai). Anti‐human C/EBPα antibody was obtained from Cell Signaling Technology (Beverly, MA, USA). Annexin V‐FITC Apoptosis Detection Kit, ROS Assay Kit‐Highly Sensitive DCFH‐DA, and Cell Cycle Analysis Kit were obtained from Dojindo Chemical Technology (Beijing). Nucleoprotein Extraction Kit was obtained from Sangon Biotech (Shanghai). Epigenase HDAC Activity/Inhibition Direct Assay Kit was obtained from Epigentek (NY, USA). Anti‐human CD11b‐FITC, anti‐human CD14‐FITC, anti‐human CD15‐FITC, anti‐human CD117 (C‐KIT）‐PE, and purified anti‐human CD49d antibodies were obtained from Biolegend (San Diego, CA, USA). Anti‐human CD45‐APC antibody, anti‐human C‐KIT, and anti‐human Histone H3 (acetyl K9) antibodies were obtained from Abcam (Cambridge, UK). Anti‐human RUNX1 antibody，anti‐human PU.1 antibody was obtained from Santa Cruz Biotechnology (CA, USA). Panobinostat, mitochondrial membrane potential assay kit was obtained from Beyotime (Shanghai). Anti‐β‐actin, β‐tubulin antibody, HRP‐Goat anti‐mouse IgG, and HRP‐Goat anti‐Rabbit IgG were obtained from ABclonal Technology (Wuhan). Selenium nanoparticles were kindly provided by Professor Tianfeng Chen (Department of Chemistry, Jinan University). Selenocysteine (Sec) probe was obtained from Changsha Yuyangyang Technology Co., Ltd. (Changsha, China).

### Plasmid Construction, Protein Expression, and Purification of V9‐HFn

Using the strategy of gene fusion expression, a short peptide containing nine amino acids (CPLDIDFYC) reported by Jäger et al.^[^ ^27^ ^]^ was displayed at the N‐terminal of HFn through a linker (GGGGS). After gene synthesis, the target gene V9‐HFn was ligated into the *Nde* I/*Bam*H I‐digested pET22b vector. Then, the recombinant V9‐HFn‐pET22b plasmid was transformed into the *E. coli* BL21 (trans Gen) competent strain. The transformed bacterial solution was cultured on an ampicillin‐containing LB plate overnight, and single clones were picked for amplification. One of the selected clones was grown overnight in ampicillin‐containing LB broth at 37 °C, 220 rpm. After that, the target gene expression was induced with 1 mm IPTG at 25 °C and 200 rpm for 8 h. Bacterial cultures were collected by centrifugation at 6000 rpm for 30 min and resuspended in 50 mm Tris‐HCl, pH 8.0 buffer. After resuspension, bacterial cells were disrupted using high‐pressure homogenization. Then, the bacterial debris was removed by centrifugation at 12 000 rpm for 20 min. The purification process was initiated by heat treating the supernatant at 72 °C for 15 min. After heat treatment, the precipitate was removed by centrifugation at 12 000 rpm for 40 min. The resulting supernatant was collected and filtered through a 0.22 µm filter and adjusted to a high salt solution containing 0.75 m NaCl. V9‐HFn was then further purified by hydrophobic chromatography followed by Superdex200 10/300 GL gel filtration. Taking bovine serum albumin as the standard, the concentration of the obtained V9‐HFn was determined by the BCA protein detection kit.

### Preparation and Characterization of V9‐HFn‐Se Nanoparticle

The V9‐HFn‐Se samples were prepared by in situ synthesis of selenium nanoparticles in the V9‐HFn cavity. In general, 2 mm sodium selenite was added to a solution of V9‐HFn (1 µm) in 50 mm Tris HCl and incubated for 6 h at room temperature with stirring. Then, sodium borohydride was added to the synthesis solution and incubated for 3 h with stirring at 4 °C. The supernatant containing the synthetic product was collected after centrifugation at 12 000 rpm and finally purified via Hiprep 26/10 desalting chromatography and Superdex200 10/300 GL gel filtration.

The microscopic morphology of the samples was characterized by TEM. In general, V9‐HFn‐Se samples (0.1 mg mL^−1^) were dropped onto copper grids and left to stand for 1 min, then negatively stained with 1% uranyl acetate. Finally, imaging was performed with a JEM‐1400 80 kV TEM (JEOL, Japan). Moreover, the metal core of V9‐HFn‐Se was characterized by the same method without negative staining. The content of Se in V9‐HFn‐Se was measured by ICP‐MS (ICPMS7800, Agilent, USA).

### Cell Culture

Kasumi‐1 cells were cultured in RPMI‐1640 medium with 20% fetal calf serum (FCS). K562, U937, NB4, MV4‐11, A549 cells were cultured in RPMI‐1640 medium with 10% FCS. HT29, U87, PC3, and CNE1 cells were cultured in DMEM medium with 10% FCS. SKNO‐1 cells were cultured in RPMI‐1640 medium with 10% FCS, 10 ng mL^−1^ recombinant human granulocyte‐macrophage colony‐stimulating factor. Kasumi‐1 was obtained from Meisen CTCC (Zhejiang, China), and SKNO‐1 was obtained from CellCook Biotech (Guangzhou, China). Fresh primary leukemia cells were obtained from t(8;21) AML patient bone marrow samples by ficoll isolation method. Human peripheral blood mononuclear cells were obtained from healthy donor peripheral blood samples by the ficoll isolation method. Ethics Committee approval was obtained from the Institutional Ethics Committee of Peking University International Hospital to the commencement of the experiments involving human samples (approval number: 2021–027 (BMR)).

### SiRNA Synthesis and Transfection

ITGA4 siRNA was synthesized by Sangon Biotech (Shanghai). Twenty‐four hours before transfection, Kasumi‐1 cells were cultured in RPMI‐1640 medium with 20% FCS without penicillin and streptomycin. Subsequently, the siRNA was mixed with the cells and incubated in a cell incubator for 5 min. Then, the cell mixture was transferred to an electric rotating cup, and the electrical transfer was performed using a Gene‐Pulser II instrument (Bio‐Rad).

### Western Blot Analysis

Cells were collected and lysed with RIPA Lysis Buffer (50 mm Tris‐HCl, 150 mm NaCl, 1% NP‐40, 0.5% sodium deoxycholate, pH 7.4) for 40 min at 4 °C. Protein extracts were run on 10−12% SDS‐PAGE and subsequently transferred to a nitrocellulose (NC) membrane. The protein‐loaded NC membrane was then blocked with 5% skim milk in PBS for 1 h at room temperature. Afterward, the NC membrane was incubated with primary antibody overnight at 4 °C. Then, the membrane was washed with PBST three times and incubated with HRP–conjugated secondary antibody for 1 h at room temperature. Finally, a chemiluminescence kit was used to detect the results. The protein intensities were quantified and represented as relative protein levels normalized to housekeeping protein.

### Flow Cytometry Analysis

Detection of the binding ability of HFn and V9‐HFn to tumor cells, 0.3 µm HFn‐FITC, or V9‐HFn‐FITC was incubated with 100 µL tumor cells in PBS containing 0.5% BSA for 45 min at 4 °C and then washed three times with PBS. Apoptosis was detected using an Annexin V‐FITC Apoptosis Detection Kit. To detect cell differentiation, cells were stained with anti‐CD11b‐FITC, anti‐CD14‐FITC, and anti‐CD15‐FITC antibodies, followed by three washes with PBS. To detect CD117 expression levels, cells were stained with specific antibodies anti‐CD117‐PE and washed with PBS three times. DCFH‐DA was used to detect ROS levels. Cells were washed using PBS three times and incubated with DCFH‐DA for 30 min at 37 °C. Mitochondrial membrane potential was detected using a mitochondrial membrane potential assay kit. The fluorescence intensity was measured by flow cytometry (BD FACSCalibur, BD Bioscience, CA).

### Morphologic Evaluation

To analyze cell morphology, Kasumi‐1 cells were continuously treated with 60 µg mL^−1^ V9‐HFn or V9‐HFn‐Se for 6 days and stained with Wright's stain after cytospin. Cell morphology was then analyzed using a microscope (Leica CS2, Hamburg, Germany).

### Quantitative Real‐time PCR

Kasumi‐1 cells were treated with 90 µg mL^−1^ V9‐HFn or V9‐HFn‐Se for 48 h. Afterward, cells were harvested and total RNA was extracted with Trizol Reagent. Using the extracted RNA as a template, cDNA was synthesized using viral reverse transcriptase. The qPCR amplification reaction was performed using ChamQ Universal SYBR qPCR Master Mix. β‐actin was also amplified as an internal reference control. The primers used are listed in Table S1 (Supporting Information).

### RNA Sequencing (RNA‐seq)

Kasumi‐1 cells were inoculated in 10 cm cell culture dishes at a density of 2 × 10^5^ mL^−1^ and then treated with 90 µg mL^−1^ V9‐HFn or V9‐HFn‐Se for 72 h. After 72 h, the cells were centrifuged, washed with PBS, and then 1 mL of Trizol reagent was added. Total RNA extraction, sequencing, and bioinformatics analysis were performed by OE Biotechnology Co., Ltd. (Shanghai, China).

### Histone Deacetylase Activity Assay

After Kasumi‐1 cells were treated with 90 µg mL^−1^ V9‐HFn or V9‐HFn‐Se for 72 h, the cells were collected, lysed, and extracted nuclear proteins using Nucleoprotein Extraction Kit. HDAC activity was detected according to the Epigenase HDAC Activity/Inhibition Direct Assay Kit.

### Selenocysteine Fluorescent Probes Analysis

Kasumi‐1 cells were subjected to a 48 h treatment with 30 µg mL^−1^ of V9‐HFn‐Se. Subsequently, the cells were carefully rinsed three times with PBS. Following this, the cells underwent a 30‐min incubation at 37 °C with a 20 µm Sec probe. Post‐incubation, the cells were washed thrice with PBS and subsequently subjected to analysis via flow cytometry.

### Targeting and Differentiation Analysis in Simulated In Vivo Microenvironment

The Balb/C mice aged 6–8 weeks old were obtained from SPF (Beijing) Biotechnology Co., Ltd. (Beijing, China). All animal experiments were approved by the Institutional Animal Care and Use Committee of the Institute of Biophysics, Chinese Academy of Sciences (approval number: SYXK2019021). To simulate the in vivo microenvironment of leukemia, normal blood cells were collected from Balb/C mice, lysed to remove the red blood cells, washed with PBS, and mixed with Kasumi‐1 cells. The obtained cell mixture was then incubated with anti‐human CD45‐APC antibody together with 0.3 µm LFn‐FITC, HFn‐FITC, or V9‐HFn‐FITC. The targeting ability of the samples to Kasumi‐1 cells was then analyzed by flow cytometry. For evaluating the therapeutic efficacy, the cell mixture was treated with 0.36 µm V9‐HFn, HFn‐Se, or V9‐HFn‐Se for 72 h and then incubated with anti‐human CD45‐APC antibody, anti‐human CD11b‐FITC antibody, and the differentiation was detected by flow cytometry.

### Statistical Analysis

All data were expressed as the mean ± standard deviation. The significance of the data was analyzed according to unpaired student's two‐sided *t*‐test, one‐way analysis of variance (ANOVA) with Dunnett's, or Tukey's test in GraphPad Prism 8 software: ^*^ *p* < 0.05, ^**^ *p* < 0.01, ^***^ *p* < 0.001, ^****^ *p* < 0.0001; ns: not significant.

## Conflict of Interest

The authors declare no conflict of interest.

## Supporting information

Supporting Information

## Data Availability

The data that support the findings of this study are available from the corresponding author upon reasonable request.

## References

[1] a) L. M. Kelly , D. G. Gilliland , Ann. Rev. Genomics Hum. Genet. 2002, 3, 179; 12194988 10.1146/annurev.genom.3.032802.115046

[2] L. F. Peterson , D.‐E. Zhang , Oncogene 2004, 23, 4255. 15156181 10.1038/sj.onc.1207727

[3] N. Martinez‐Soria , L. McKenzie , J. Draper , A. Ptasinska , H. Issa , S. Potluri , H. J. Blair , A. Pickin , A. Isa , P. S. Chin , R. Tirtakusuma , D. Coleman , S. Nakjang , S. Assi , V. Forster , M. Reza , E. Law , P. Berry , D. Mueller , C. Osborne , A. Elder , S. N. Bomken , D. Pal , J. M. Allan , G. J. Veal , P. N. Cockerill , C. Wichmann , J. Vormoor , G. Lacaud , C. Bonifer , et al., Cancer Cell 2018, 34, 626. 30300583 10.1016/j.ccell.2018.08.015 PMC6179967

[4] T. Pabst , B. U. Mueller , N. Harakawa , C. Schoch , T. Haferlach , G. Behre , W. Hiddemann , D.‐E. Zhang , D. G. Tenen , Nat. Med. 2001, 7, 444. 11283671 10.1038/86515

[5] R. K. Vangala , M. S. Heiss‐Neumann , J. S. Rangatia , S. M. Singh , C. Schoch , D. G. Tenen , W. Hiddemann , G. Behre , Blood 2003, 101, 270. 12393465 10.1182/blood-2002-04-1288

[6] Y.‐Y. Wang , G.‐B. Zhou , T. Yin , B. Chen , J.‐Y. Shi , W.‐X. Liang , X.‐L. Jin , J.‐H. You , G. Yang , Z.‐X. Shen , J. Chen , S.‐M. Xiong , G.‐Q. Chen , F. Xu , Y.‐W. Liu , Z. Chen , S.‐J. Chen , Proc. Natl. Acad. Sci. U. S. A. 2005, 102, 1104. 15650049 10.1073/pnas.0408831102 PMC545849

[7] K. Lam , D.‐E. Zhang , Front. Biosci. 2012, 17, 1120. 10.2741/3977 PMC3433167 22201794

[8] a) G.‐Q. Chen , X.‐G. Shi , W. Tang , S.‐M. Xiong , J. Zhu , X. Cai , Z.‐G. Han , J.‐H. Ni , G.‐Y. Shi , P.‐M. Jia , M.‐M. Liu , K.‐L. He , C. Niu , J. Ma , P. Zhang , T.‐D. Zhang , P. Paul , T. Naoe , K. Kitamura , W. Miller , S. Waxman , Z.‐Y. Wang , H. de The , S.‐J. Chen , Z. Chen , Blood 1997, 89, 3345; 9129041

[9] M. P. Rayman , Lancet 2012, 379, 1256. 22381456 10.1016/S0140-6736(11)61452-9

[10] A. Khurana , S. Tekula , M. A. Saifi , P. Venkatesh , C. Godugu , Biomed. Pharmacother. 2019, 111, 802. 30616079 10.1016/j.biopha.2018.12.146

[11] Y. Jin , L. Cai , Q. Yang , Z. Luo , L. Liang , Y. Liang , B. Wu , L. Ding , D. Zhang , X. Xu , L. Zhang , F. Zhou , Carbohydr. Polym. 2020, 240, 116329. 32475588 10.1016/j.carbpol.2020.116329

[12] a) G. Zhao , X. Wu , P. Chen , L. Zhang , C. S. Yang , J. Zhang , Free Radical Biol. Med. 2018, 126, 55; 30056082 10.1016/j.freeradbiomed.2018.07.017

[13] Y. Huang , E. Su , J. Ren , X. Qu , Nano Today 2021, 38, 101205.

[14] B. Jiang , L. Fang , K. Wu , X. Yan , K. Fan , Theranostics 2020, 10, 687. 31903145 10.7150/thno.39827 PMC6929972

[15] a) C. Wang , W. Zhang , Y. He , Z. Gao , L. Liu , S. Yu , Y. Hu , S. Wang , C. Zhao , H. Li , J. Shi , W. Zhou , F. Li , H. Yue , Y. Li , W. Wei , G. Ma , D. Ma , Nat. Nanotechnol. 2021, 16, 1413; 34697490 10.1038/s41565-021-00980-7

[16] a) Q. Liu , J. Tian , J. Liu , M. Zhu , Z. Gao , X. Hu , A. C. Midgley , J. Wu , X. Wang , D. Kong , J. Zhuang , J. Liu , X. Yan , X. Huang , Adv. Mater. 2021, 33, 2103128; 10.1002/adma.202103128 34350648

[17] a) T. Matsunaga , N. Takemoto , T. Sato , R. Takimoto , I. Tanaka , A. Fujimi , T. Akiyama , H. Kuroda , Y. Kawano , M. Kobune , J. Kato , Y. Hirayama , S. Sakamaki , K. Kohda , K. Miyake , Y. Niitsu , Nat. Med. 2003, 9, 1158; 12897778 10.1038/nm909

[18] H. Döhner , E. Estey , D. Grimwade , S. Amadori , F. R. Appelbaum , T. Büchner , H. Dombret , B. L. Ebert , P. Fenaux , R. A. Larson , R. L. Levine , F. Lo‐Coco , T. Naoe , D. Niederwieser , G. J. Ossenkoppele , M. Sanz , J. Sierra , M. S. Tallman , H.‐F. Tien , A. H. Wei , B. Löwenberg , C. D. Bloomfield , Blood 2017, 129, 424. 27895058 10.1182/blood-2016-08-733196 PMC5291965

[19] a) Y. Lu , Z. G. Peng , T. T. Yuan , Q. Q. Yin , L. Xia , G. Q. Chen , Leukemia 2008, 22, 378; 17989718 10.1038/sj.leu.2405020

[20] K. R. Stengel , J. D. Ellis , C. L. Spielman , M. L. Bomber , S. W. Hiebert , Mol. Cell 2021, 81, 530. 33382982 10.1016/j.molcel.2020.12.005 PMC7867650

[21] a) J. Wang , Y. Saunthararajah , R. L. Redner , J. M. Liu , Cancer Res. 1999, 59, 2766; 10383127

[22] W. Feng , M. Li , Y. Sun , G. Feng , Anal. Chem. 2017, 89, 6106. 28504517 10.1021/acs.analchem.7b00824

[23] D. J. Pearce , D. Taussig , K. Zibara , L.‐L. Smith , C. M. Ridler , C. Preudhomme , B. D. Young , A. Z. Rohatiner , T. A. Lister , D. Bonnet , Blood 2006, 107, 1166. 16234360 10.1182/blood-2005-06-2325 PMC1895911

[24] G. Yang , M. A. Thompson , S. J. Brandt , S. W. Hiebert , Oncogene 2007, 26, 91. 16799637 10.1038/sj.onc.1209760

[25] a) V. Gelmetti , J. Zhang , M. Fanelli , S. Minucci , P. G. Pelicci , M. A. Lazar , Mol. Cell. Biol. 1998, 18, 7185; 9819405 10.1128/mcb.18.12.7185 PMC109300

[26] T. Liu , L. Xu , L. He , J. Zhao , Z. Zhang , Q. Chen , T. Chen , Nano Today 2020, 35, 100975.

[27] S. Jäger , A. Jahnke , T. Wilmes , S. Adebahr , F. N. Vögtle , E. deLima‐Hahn , D. Pfeifer , T. Berg , M. Lübbert , M. Trepel , Leukemia 2007, 21, 411. 17252013 10.1038/sj.leu.2404548

