# Supplementary material for: Protein‐Nanocaged Selenium Induces t(8;21) Leukemia Cell Differentiation via Epigenetic Regulation
Source: Adv Sci (Weinh). 2023 Oct 27;10(35):2300698. doi: 10.1002/advs.202300698 (PMC10724402; doi:10.1002/advs.202300698)
Supplement: Supplementary file 1 — Supporting Information [file ADVS-10-2300698-s001.pdf]

ADVANCED SCIENCE
Open Access

Supporting Information

for *Adv. Sci.*, DOI 10.1002/advs.202300698

Protein-Nanocaged Selenium Induces t(8;21) Leukemia Cell Differentiation via Epigenetic Regulation

Long Fang, Ruofei Zhang, Lin Shi, Jiaying Xie, Long Ma, Yili Yang, Xiyun Yan and Kelong Fan**

Supporting Information

Protein-Nanocaged Selenium Induces t(8;21) Leukemia Cell Differentiation via Epigenetic Regulation

Long Fang[1,2]†, Ruofei Zhang[2]†, Lin Shi[3]†, Jiaying Xie[2], Long Ma[2], Yili Yang[4], Xiyun Yan[2,5], and Kelong Fan[2,5]**

[1] Savaid Medical School, University of Chinese Academy of Sciences, Beijing 100049, China.

[2] CAS Engineering Laboratory for Nanozyme, Key Laboratory of Protein and Peptide Pharmaceutical, Institute of Biophysics, Chinese Academy of Sciences, Beijing 100101, China.

[3] Department of Hematology, Peking University International Hospital, Beijing 102206, China.

[4] China Regional Research Centre, International Centre of Genetic Engineering and Biotechnology, Taizhou 212200, China.

[5] Nanozyme Medical Center, School of Basic Medical Sciences, Zhengzhou University, Zhengzhou 450052, China.

*Corresponding author

E-mail: yanxy@ibp.ac.cn (X. Yan), and fankelong@ibp.ac.cn (K. Fan).

†These authors contributed equally to this work.

This PDF file includes:

Figures S1 to S18

Supplementary Tables 1

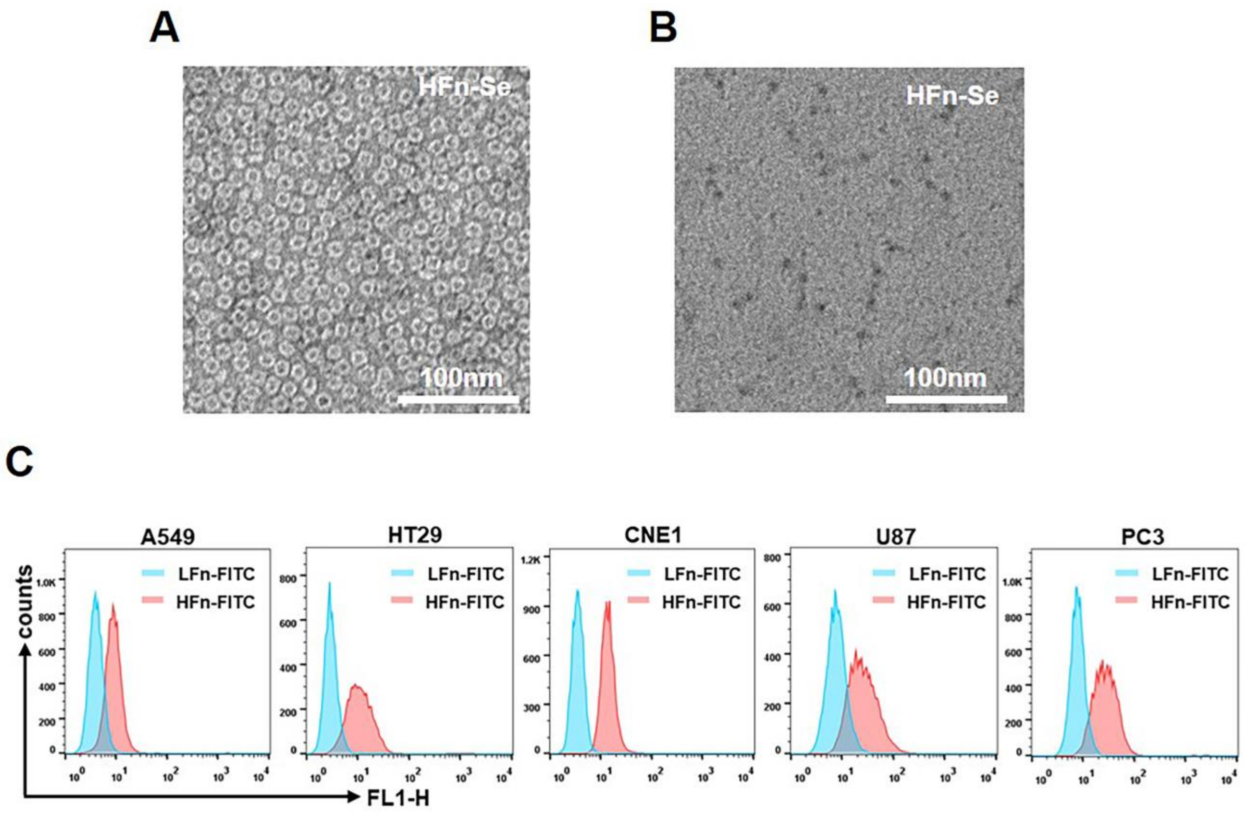

Figure S1. TEM images of (A) the ferritin shell and (B) the selenium core of HFn-Se. Scale bar = 100 nm. C. Binding capacity of HFn on different solid tumor cells.

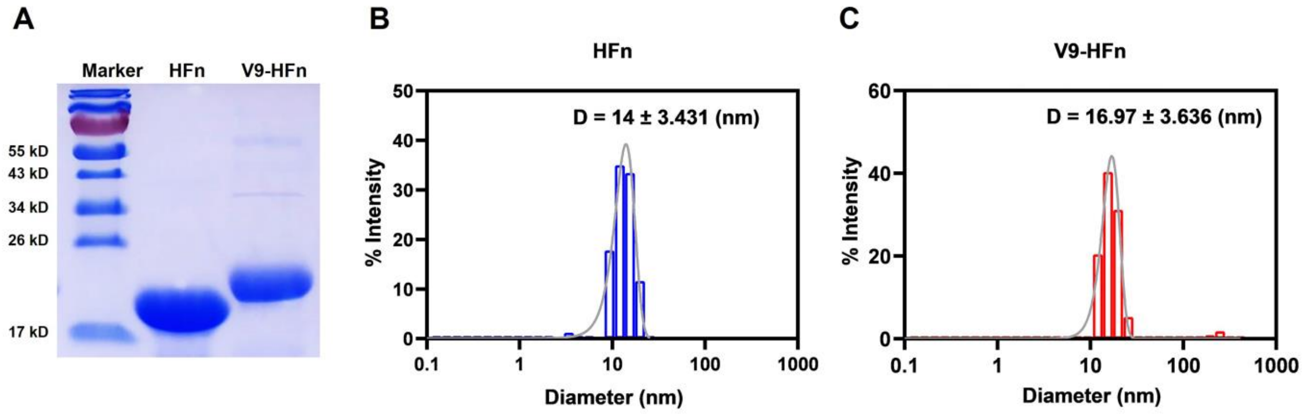

Figure S2. A. The SDS-PAGE analysis of HFn and V9-HFn. The DLS analysis of HFn (B) and V9-HFn (C).

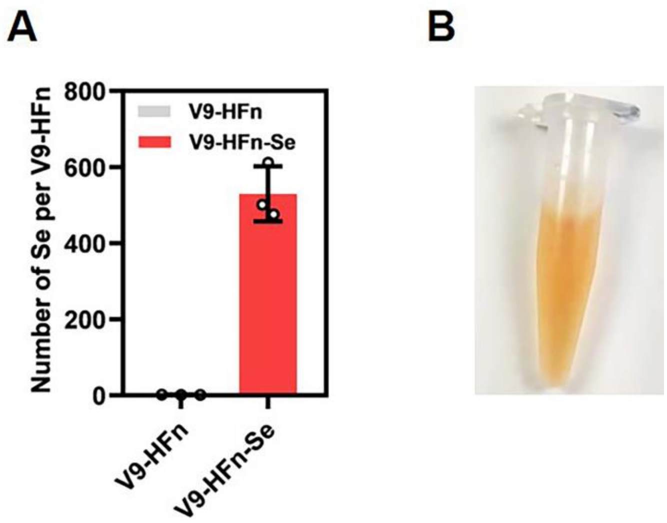

Figure S3. The characterization of V9-HFn-Se. A. Analysis of selenium content in V9-HFn-Se by ICP-MS (n = 3). B. Images of V9-HFn-Se in PBS buffer. The data represents mean ± SD.

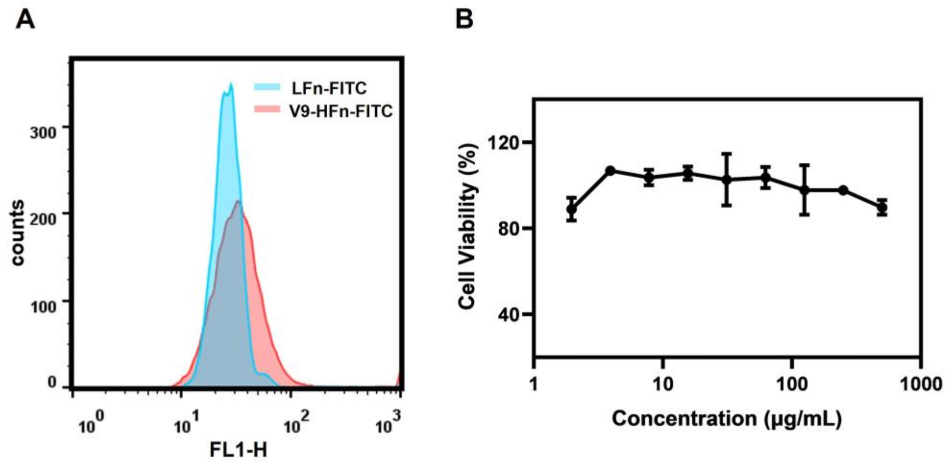

Figure S4. The binding and cytotoxic effect of V9-HFn to normal cells. A. The targeted binding analysis of V9-HFn to HUVECs cells. Light chain ferritin (LFn) was chosen as a negative control. B. Cell viability analysis of HUVECs cells treated with V9-HFn (n = 3).The data represents mean ± SD.

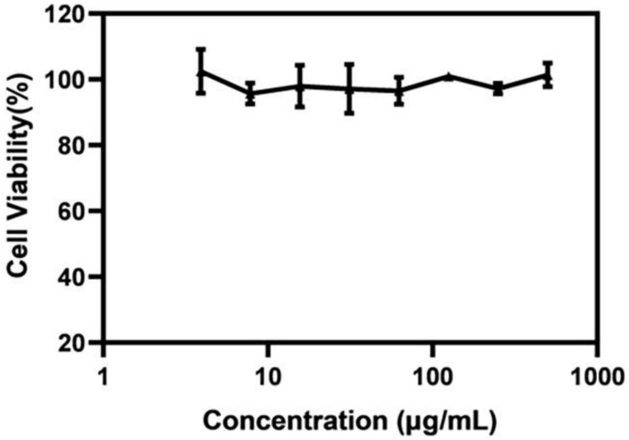

Figure S5. The effect of V9-HFn on the viability of Kasumi-1 cells (n = 3).The data represents mean ± SD..

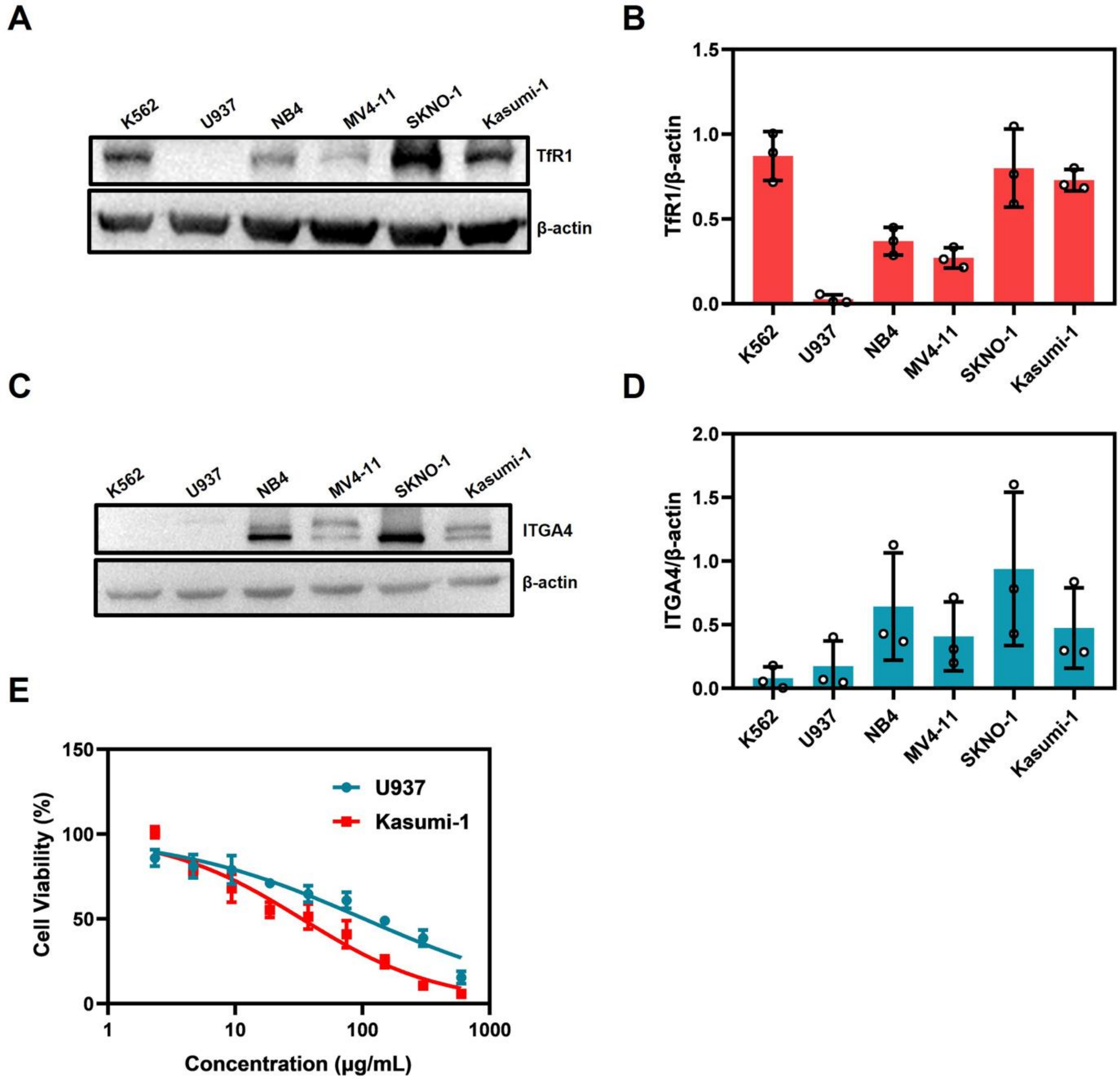

Figure S6. Immunoblotting was performed to assess the protein expression levels of TfR1 (A) and ITGA4 (C) in various leukemia cells. Quantification of the immunoblot signals was conducted to determine the relative abundance of TfR1 (B) and ITGA4 (D) proteins in the different cell lines (n = 3). Protein intensity of TfR1 and ITGA4 were quantified and represented as relative protein levels normalized to β-actin. E. Cell viability analysis of Kasumi-1 and U937 cells treated with V9-HFn-Se (n = 3). The data represent mean ± SD.

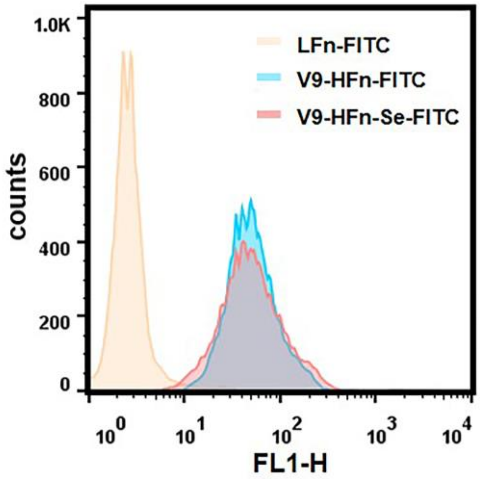

Figure S7. The targeted binding analysis of V9-HFn-Se and V9-HFn to Kasumi-1 cells.

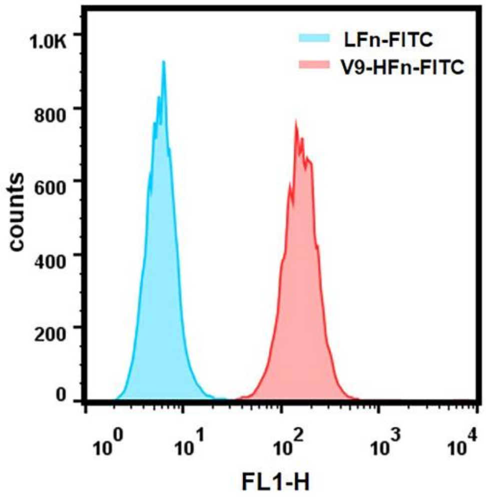

Figure S8. The targeted binding analysis of V9-HFn to SKNO-1 cells. Light chain ferritin (LFn) was chosen as a negative control.

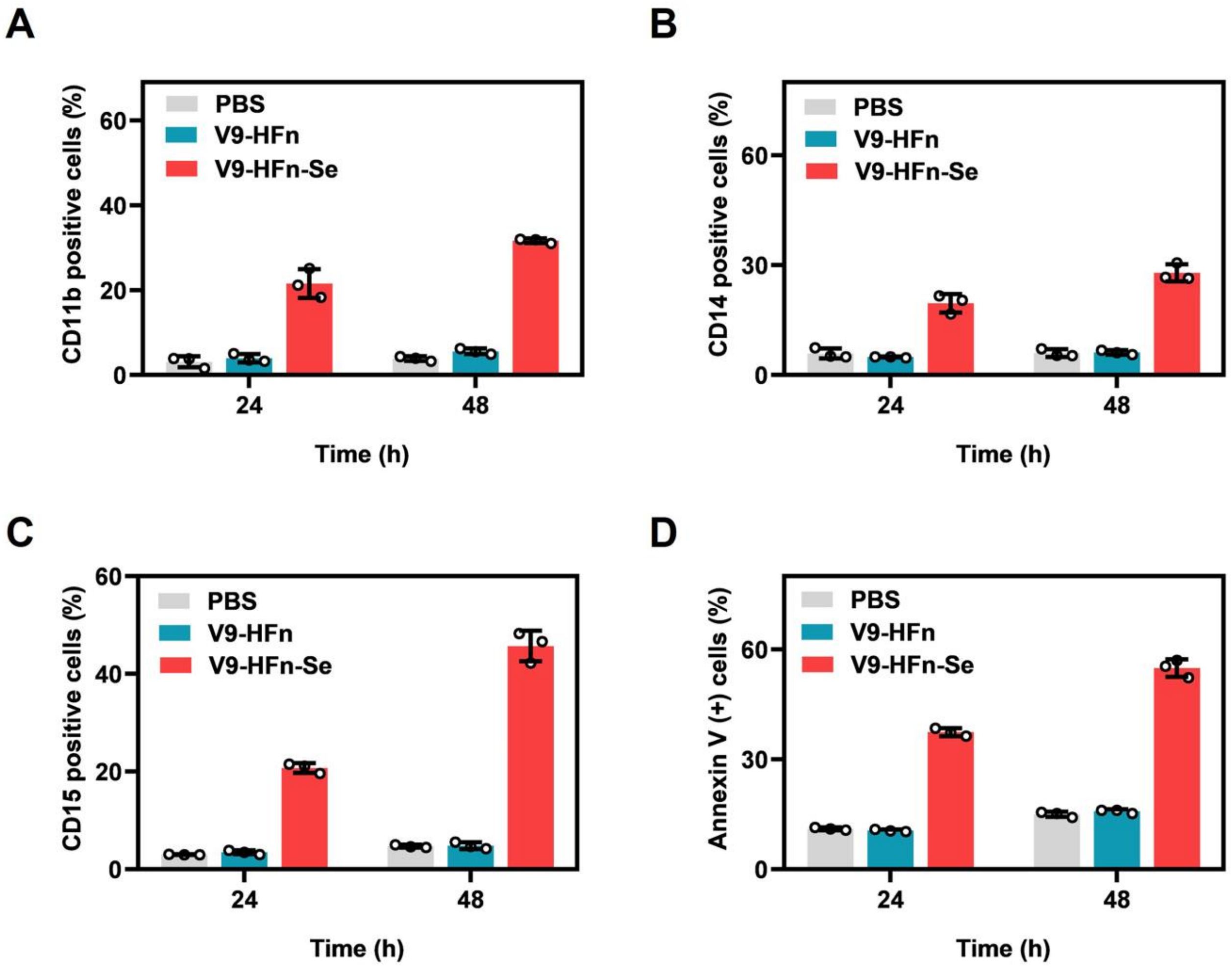

Figure S9. Effects of V9-HFn-Se on differentiation and apoptosis of SKNO-1 cells. A-C. Flow cytometric analysis of CD11b, CD14, and CD15 expression in SKNO-1 cells treated with V9-HFn-Se in a time-dependent manner (n = 3). D. Expression of Annexin-V on SKNO-1 cells treated with V9-HFn-Se in a time-dependent manner (n = 3).

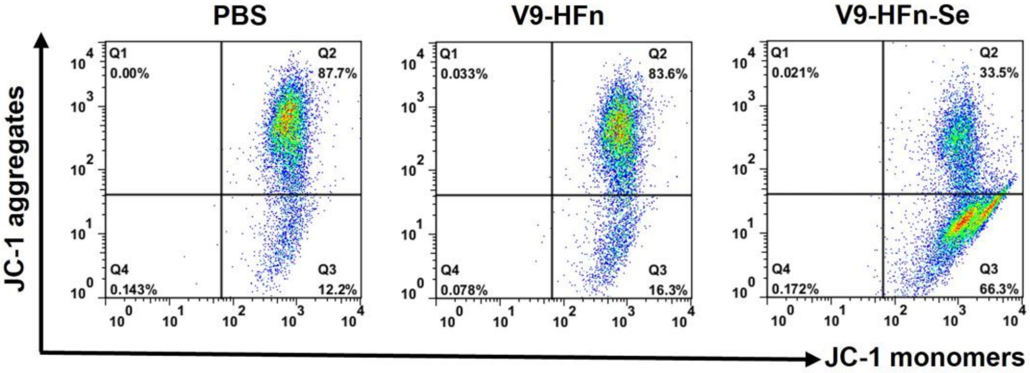

Figure S10. Effects of V9-HFn-Se on the mitochondrial membrane potential of Kasumi-1 cells.

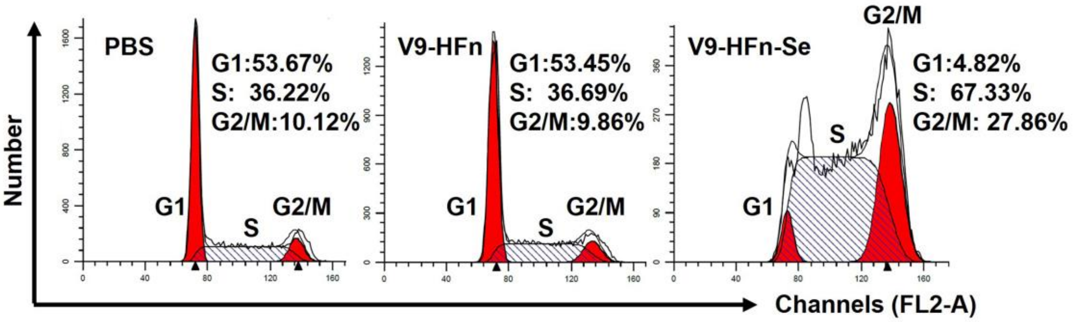

Figure S11. Effects of V9-HFn-Se on the cell cycle of Kasumi-1 cells.

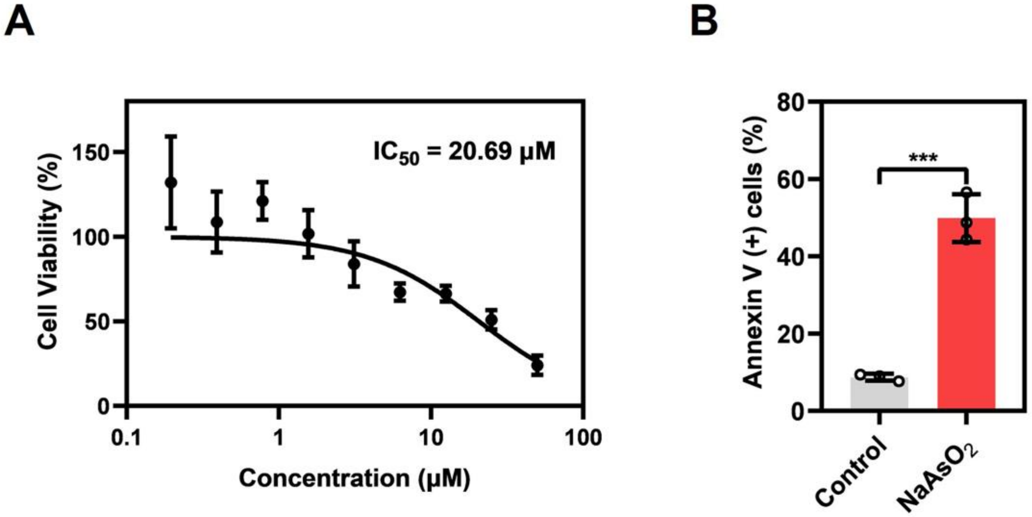

Figure S12. Cell cytotoxicity analyses of $NaAsO_2$. A. Cell viability analysis in Kasumi-1 cells after treatment with different concentrations of $NaAsO_2$. B. The ratio of Annexin V-positive Kasumi-1 cells treated with 50 µM $NaAsO_2$ for 48 hours (n = 3). ***$p < 0.001$. Two-tailed unpaired Student's t-test was performed. The data represent mean ± SD.

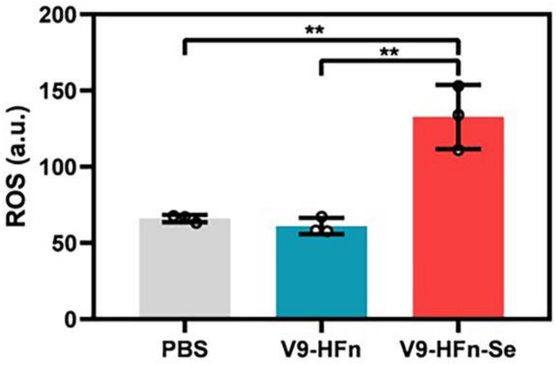

Figure S13. Flow cytometric analysis of ROS levels in Kasumi-1 cells treated with 90 µg/mL V9-HFn-Se for 24 hours (n = 3). **$p < 0.01$. One-way ANOVA with Tukey's test was performed. The data represent mean ± SD.

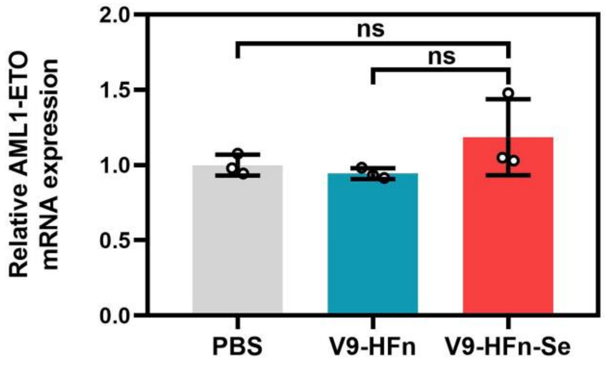

Figure S14. The qPCR analysis shows the level of AML1-ETO in Kasumi-1 cells treated with 90 μg/mL V9-HFn-Se for 48 hours (n = 3). ns: not significant. One-way ANOVA with Tukey's test was performed. The data represents mean ± SD.

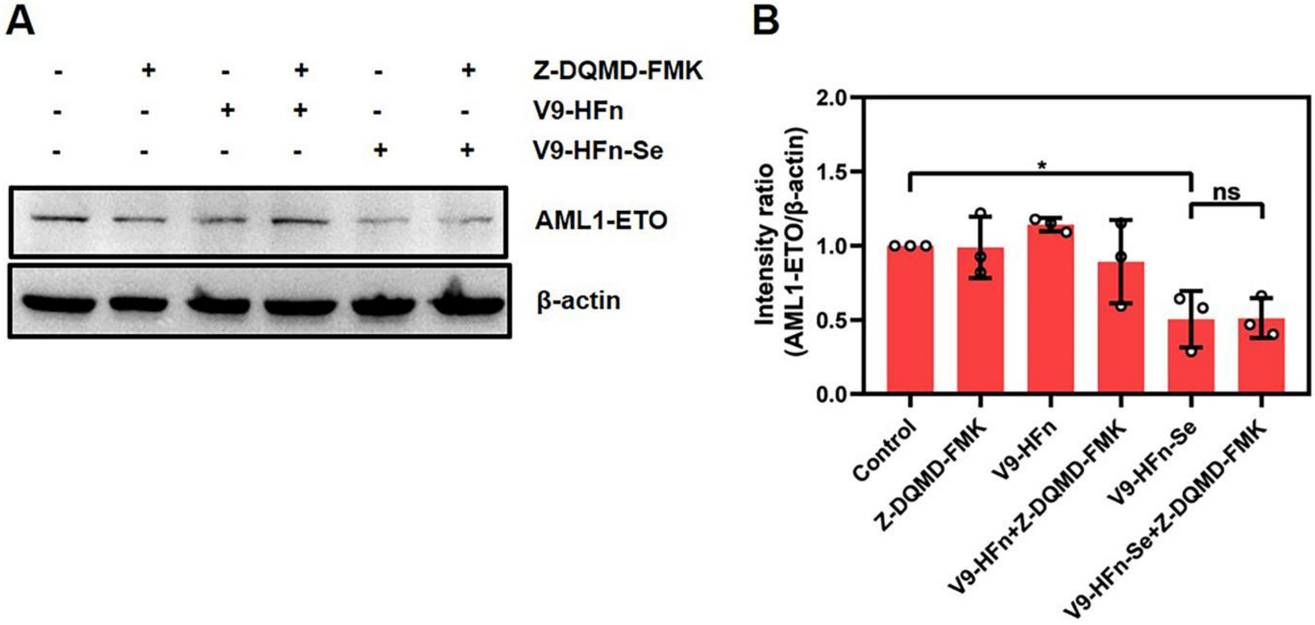

Figure S15. (A) Immunoblotting and (B) quantification of AML1-ETO level in V9-HFn-Se-treated Kasumi-1 cells in the presence or absence of caspase-3 inhibitor (Z-DQMD-FMK) pretreatment (n = 3). Protein intensity of AML1-ETO was quantified and represented as relative protein levels normalized to β-actin. *$p < 0.05$. One-way ANOVA with Tukey's test was performed. The data represent mean ± SD.

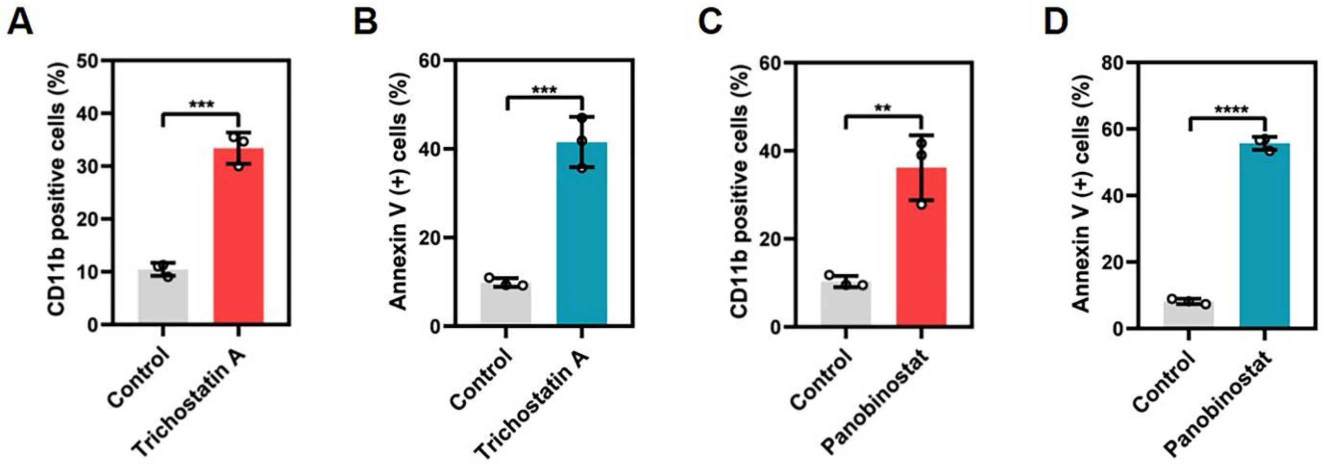

Figure S16. Effects of histone deacetylase inhibitors on differentiation and apoptosis of Kasumi-1 cells. A. Flow cytometric analysis of CD11b expression in Kasumi-1 cells treated with 4 μM trichostatin A for 48 hours (n = 3). B. Expression of Annexin-V on Kasumi-1 cells treated with 4 μM trichostatin A for 48 hours (n = 3). C. Flow cytometric analysis of CD11b expression in Kasumi-1 cells treated with 5 nM panobinostat for 48 hours (n = 3). D. Expression of Annexin-V on Kasumi-1 cells treated with 5 nM panobinostat for 48 hours (n = 3). **p < 0.01, ***p < 0.001, ****p < 0.0001. Two-tailed unpaired Student's t-test was performed. The data represent mean ± SD.

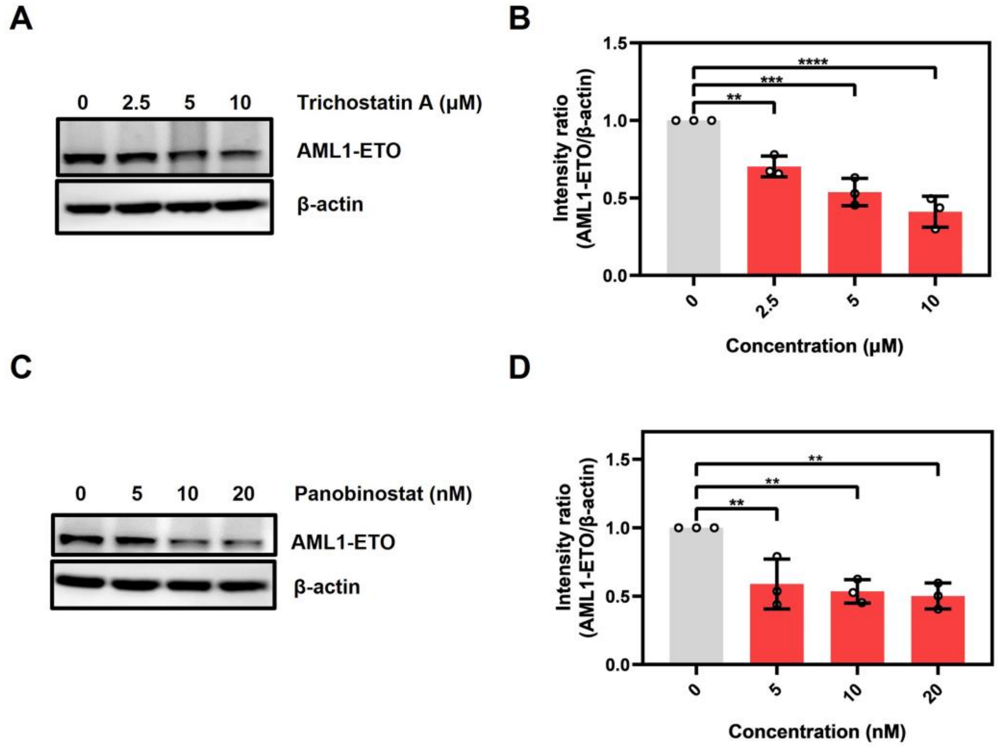

Figure S17. Histone deacetylase inhibitors decreased AML1-ETO protein level in Kasumi-1 cells. (A) Immunoblotting and (B) quantification of AML1-ETO protein level in Kasumi-1 cells treated with different concentrations of trichostatin A for 24 hours (n=3). Protein intensity of AML1-ETO was quantified and represented as relative protein levels normalized to β-actin. (C) Immunoblotting and (D) quantification of AML1-ETO protein level in Kasumi-1 cells treated with different concentrations of panobinostat for 24 hours (n = 3). Protein intensity of AML1-ETO was quantified and represented as relative protein levels normalized to β-actin. **$p < 0.01$, ***$p < 0.001$, ****$p < 0.0001$. One-way ANOVA with Dunnett's test (B, D) was performed. The data represent mean ± SD.

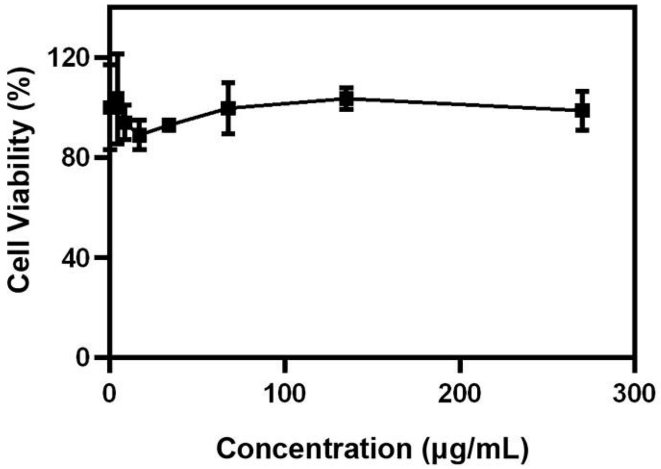

Figure S18. Cell viability analysis in human peripheral blood mononuclear cells treated with V9-HFn-Se (n = 3). The data represent mean ± SD.

Supplementary Table 1. qPCR primers list

Primer name	Sequence (5’-3’)	
CD82	GCTCATTCGAGACTACAACAGC	Forward
CD82	GTGACCTCAGGGCGATTCA	Reverse
UBQLN1	ACAAGAGCAGTTTGGTGGTA	Forward
UBQLN1	GCTGATGAACTCTGGGAAGT	Reverse
AML1-ETO	CACAAACCCACCGCAAGTC	Forward
AML1-ETO	TGGAGTGCTTCTCAGTACGATTTC	Reverse
IL-3	TTAAAGCAGCCACCTTTGCC	Forward
IL-3	TTGAATGCCTCCAGGTTTGG	Reverse
